# A Case of Severe Respiratory Failure Caused by Metapneumovirus and Influenza Virus in a Patient with HIV Infection

**DOI:** 10.3390/v17030289

**Published:** 2025-02-20

**Authors:** Luca Pipitò, Chiara Vincenza Mazzola, Eleonora Bono, Claudia Gioè, Giovanni M. Giammanco, Celestino Bonura, Antonio Cascio

**Affiliations:** 1 Department of Health Promotion, Mother and Child Care, Internal Medicine and Medical Specialties “G. D’Alessandro”, University of Palermo, 90133 Palermo, Italy; luca.pipito@community.unipa.it (L.P.); chiaravincenza.mazzola@community.unipa.it (C.V.M.); eleonora.bono@community.unipa.it (E.B.); giovanni.giammanco@unipa.it (G.M.G.); 2 Infectious and Tropical Disease Unit, AOU Policlinico “P. Giaccone”, 90133 Palermo, Italy; claudia.gioe@policlinico.pa.it; 3 Microbiology and Virology Unit, AOU Policlinico “P. Giaccone”, 90133 Palermo, Italy

**Keywords:** human metapneumovirus, HMPV, influenza A virus, HIV infection, severe pneumonia, molecular diagnostics, multiplex PCR, antimicrobial stewardship, community-acquired pneumonia (CAP)

## Abstract

Background: Human metapneumovirus (HMPV) is a significant cause of respiratory infections, particularly in children, the elderly, and immunocompromised individuals. However, data on HMPV infection in people living with HIV (PLWH) are limited, and cases of co-infection with influenza A virus in this population have not been previously described. Case Presentation: We reported the case of a 73-year-old HIV-positive man with multiple comorbidities, including insulin-dependent diabetes mellitus, who presented with fever, asthenia, and glycometabolic decompensation. Despite an initially unremarkable chest computed tomography (CT) scan, the patient developed progressive respiratory failure, requiring high-flow oxygen therapy. Molecular testing using the BIOFIRE^®^ FILMARRAY^®^ Pneumonia Panel Plus identified HMPV and influenza A virus as the causative pathogens. Bacterial cultures were negative, allowing for the discontinuation of empirical antibiotic therapy. The patient was successfully weaned off oxygen therapy and discharged after clinical improvement. Conclusions: This case highlights the potential severity of HMPV and influenza A co-infection in PLWH, emphasizing the importance of molecular diagnostics in distinguishing viral from bacterial infections. Rapid and accurate pathogen identification is essential for guiding appropriate antimicrobial stewardship and optimizing patient outcomes in community-acquired pneumonia.

## 1. Introduction

The ongoing surge in human metapneumovirus (HMPV) cases in China has raised significant global concerns regarding its potential to cause severe complications, particularly in vulnerable populations, including children, the elderly, and immunocompromised individuals [1,2]. Colder weather conditions and increased indoor activity facilitate the transmission of respiratory viruses. HMPV spreads primarily through respiratory droplets expelled during coughing and sneezing [1]. HMPV is an enveloped, non-segmented, negative-stranded RNA virus belonging to the subfamily *Pneumovirinae* [3]. It has been associated with acute severe respiratory syndrome and high mortality rates, particularly in patients with underlying conditions [4], including malignancies [5]. The prevalence of HMPV among hospitalized patients with acute respiratory infections has been estimated at 6.24% [6]. Clinical presentations of HMPV infection in adults vary widely. While some patients remain asymptomatic, others develop symptoms ranging from mild upper respiratory tract infections to severe pneumonia. Common manifestations include cough, nasal congestion, and dyspnea, while purulent cough, wheezing, sore throat, fever, pneumonia, bronchiolitis, conjunctivitis, and otitis media have also been reported. In rare cases, HMPV infection may present itself as a mononucleosis-like illness, even in immunocompetent adults [7]. Additionally, atypical cases that pose diagnostic challenges have been described in the literature, including pneumonia-mimicking Legionnaires’ disease [8], myocarditis [9], and encephalomyelitis [10]. The course of HMPV infection in people living with HIV (PLWH) remains poorly characterized, with only a few cases documented in the literature [11,12,13,14]. To the best of our knowledge, we present the first reported case of severe respiratory syndrome caused by co-infection with HMPV and influenza A virus in a patient with HIV infection.

## 2. Case Presentation

### 2.1. Presentation to Emergency Department

A 73-year-old man presented to the emergency department with profound asthenia and fever. He complained about these symptoms for about three days. At the emergency department he was febrile, with a body temperature of 38.5 °C. Laboratory tests revealed an elevated C-reactive protein (CRP) level of 69.7 mg/L and hyperglycemia with a blood glucose level of 391 mg/dL. Arterial blood gas (ABG) analysis showed the following values: pH—7.46, lactate—1.1 mmol/L, bicarbonate (HCO_3_^−^)—25.1 mmol/L, oxygen saturation (SpO_2_)—95.8% in room air, partial pressure of oxygen (PaO_2_)—70 mmHg, and partial pressure of carbon dioxide (PaCO_2_)—33 mmHg. A chest computed tomography (CT) scan was unremarkable but revealed a 1.5 cm air bubble in the anteromedial basal segment of the left lower lobe, fibrotic atelectatic streaks in the bilateral basal regions, and mediastinal lymphadenopathy. Brain CT demonstrated diffuse hypodensity of the periventricular white matter, which is suggestive of chronic cerebrovascular insufficiency.

### 2.2. Admission to Infectious Disease Unit

**Figure 1:**
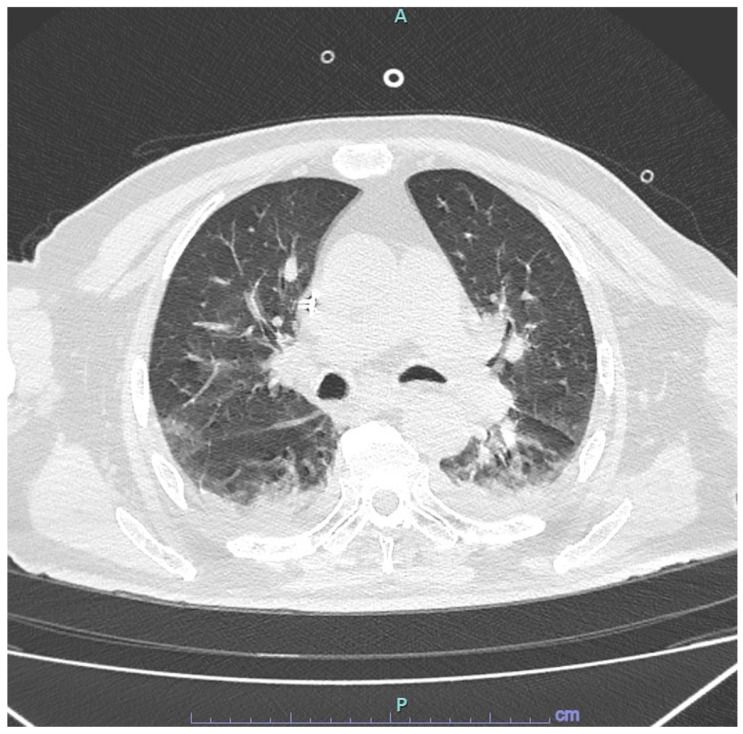
The chest CT scan shows areas of increased parenchymal density with a mixed appearance in the bilateral lower lobes, partly as ground-glass opacity and partly as consolidation, and are more pronounced on the left with involvement of the entire basal pyramid, the posterior segment of the right upper lobe, and the lingula of the left upper lobe. A: anterior; P: posterior.

The patient was admitted to the infectious diseases unit for fever associated with glycometabolic decompensation. His medical history included HIV infection, managed with darunavir/cobicistat/tenofovir alafenamide/emtricitabine, insulin-dependent diabetes mellitus, and the presence of a cardiac pacemaker. Upon admission, his vital signs were as follows: blood pressure—150/70 mmHg, heart rate—103 beats per minute, oxygen saturation—92%, body temperature—37.7 °C, and respiratory rate—26 breaths per minute. HIV infection was well-controlled, with an undetectable viral load and a CD4 T cell count of 139 cells/μL (CD4/CD8 ratio = 0.7). Initial laboratory findings upon admission to the infectious diseases unit are detailed in Table 1. Intravenous insulin therapy was initiated, and empirical antibiotic therapy with piperacillin—tazobactam was started after blood cultures were obtained. Within 24 h, the patient developed progressive dyspnea, fatigue, and a subsequent cough. ABG analysis revealed type 1 respiratory failure (PaO_2_ = 54 mmHg), prompting the initiation of supplemental oxygen therapy at 2 L/min via nasal cannula. Over the next two days, respiratory failure worsened, with an increased respiratory rate (36 breaths per minute) and a rise in CRP to 262 mg/L. Ceftaroline was added to the empirical treatment regimen to provide extended coverage for methicillin-resistant *Staphylococcus aureus* (MRSA) infection. Oxygen therapy was escalated to a Venturi mask (fraction of inspired oxygen [FiO_2_] = 40%), followed by high-flow nasal cannula (HFNC) therapy at 60 L/min. A repeat chest CT scan revealed bilateral pleural effusion and areas of increased parenchymal density with a mixed pattern, comprising both ground-glass opacities and consolidative changes in both lower lobes (Figure 1). Blood cultures remained negative.

### 2.3. Microbiological Results on Respiratory Samples

**Figure 2:**
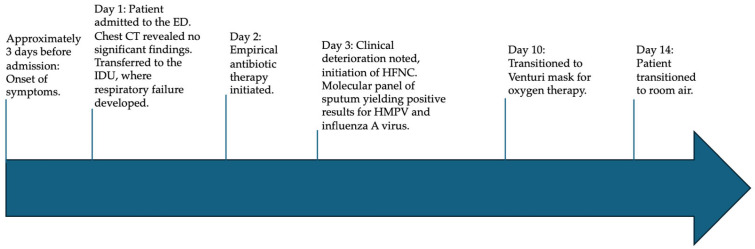
Timeline of patient’s clinical history. ED: emergency department, CT: computed tomography, IDU: infectious disease unit, HFNC: high-flow nasal cannula, HMPV: Human metapneumovirus.

On day 3, a multiplex molecular panel (BIOFIRE^®^ FILMARRAY^®^ Pneumonia Panel Plus, Salt Lake City, UT, USA), which can detect 27 bacterial (*Acinetobacter calcoaceticus–baumannii complex*, *Enterobacter cloacae complex*, *Escherichia coli*, *Haemophilus influenzae*, *Klebsiella aerogenes*, *Klebsiella oxytoca*, *Klebsiella pneumoniae* group, *Moraxella catarrhalis*, *Proteus* spp., *Pseudomonas aeruginosa*, *Serratia marcescens*, *Staphylococcus aureus*, *Streptococcus agalactiae*, *Streptococcus pneumoniae*, *Streptococcus pyogenes*, *Chlamydia pneumoniae*, *Legionella pneumophila*, *Mycoplasma pneumoniae*) and viral pathogens (adenovirus, coronavirus, HMPV, human rhinovirus/enterovirus, influenza A virus, influenza B virus, parainfluenza virus, respiratory syncytial virus) associated with pneumonia and other lower respiratory tract infections, as well as seven genetic markers of antibiotic resistance (IMP, KPC, NDM, OXA-48-like, VIM, CTX-M, mecA/C, and MREJ) [15], was performed on sputum. The test identified human metapneumovirus (HMPV) (cycle threshold = 31) and influenza A virus (cycle threshold = 33). The patient had not received the influenza vaccine. Oseltamivir was initiated for ten days, while no specific antiviral therapy was available for HMPV. Sputum cultures for bacterial pathogens remained negative, leading to the discontinuation of antibiotic therapy. After seven days, the patient was successfully weaned off HFNC therapy and continued oxygen support via a Venturi mask (FiO_2_ = 40%). Oxygen therapy was discontinued entirely after an additional five days. With the normalization of inflammatory markers, the patient was ultimately discharged to a healthcare facility for further rehabilitation. The timeline clinical history is illustrated in Figure 2.

## 3. Discussion

HMPV is a significant cause of respiratory disease, particularly in children, the elderly, and immunocompromised individuals. Recently, numerous cases have been reported in China; however, data on the course of HMPV infection in PLWH remain scarce [1,12]. Our patient presented multiple risk factors for severe viral respiratory syndrome, including advanced age, diabetes, and HIV infection. The glycometabolic decompensation was the primary reason for the patient’s admission, which accounted for the presence of neutrophilia in the initial laboratory findings. Additionally, the co-infection with HMPV and influenza A virus likely contributed to a more severe clinical presentation. To date, no other cases of HMPV and influenza A virus co-infection in PLWH have been reported in the literature. Defects in humoral and cellular immunity associated with HIV infection may influence both the progression and severity of common viral infections. While antiretroviral therapy can partially restore immune function, PLWH may remain at increased risk of respiratory morbidity, particularly if their ability to mount antigen-specific immune responses is compromised [16]. Experimental studies have shown that HMPV induces alterations in both innate and adaptive immunity in laboratory-infected mice. Specifically, it has been associated with significant changes in interferon-gamma transcription, as well as an increase in colonic CD8^+^ T cells and memory precursor effector CD8^+^ T cells. These findings suggest a potential role for HMPV in systemic immune dysregulation in infected patients [17]. Furthermore, HMPV can induce aberrant T cell responses, leading to exacerbated lung inflammation and impaired T and B cell memory immunity. This occurs through mechanisms such as immunological synapse disruption mediated by viral proteins or soluble factors and the induction of pro-inflammatory cytokines by epithelial cells. CD4^+^ and CD8^+^ T cells play a critical role in clearing HMPV from infected lungs in experimental models [18]. Likewise, an effective antiviral immune response driven by IFN-γ-secreting CD4^+^ and CD8^+^ effector and memory T cells is essential for limiting viral spread in the airways and reducing the risk of bronchiolitis and pneumonitis [18,19]. However, despite the resolution of the acute disease, long-term immunological memory of T and B cells remains poor, contributing to recurrent infections and sustained viral circulation within the community [18,19].

While previous studies suggested that HIV-positive adults without advanced immunosuppression or comorbidities do not exhibit increased susceptibility to the H1N1 influenza virus or more severe disease outcomes [20], a meta-analysis from Africa reported an increased risk of mortality in PLWH with acute respiratory tract infections, including those caused by HMPV and influenza virus, without differences in viral etiology compared to HIV-negative individuals [13]. Additionally, another study from Africa found a higher burden of HMPV-associated severe acute respiratory illness in PLWH aged 5–64 years compared to HIV-negative individuals [14]. In a prior study, Klein et al. identified viruses, including influenza and metapneumovirus, as the primary etiologic agents of febrile respiratory illness in PLWH, emphasizing the importance of viral diagnostics in guiding clinical management and reducing unnecessary antibiotic use [11]. Tran et al. reported a case of acute respiratory distress syndrome due to HMPV in an HIV-positive patient initially misdiagnosed with Pneumocystis pneumonia, requiring mechanical ventilation [12]. In our case, rapid molecular diagnostics using the BIOFIRE^®^ FILMARRAY^®^ Pneumonia Panel Plus [15] were performed on induced sputum, identifying HMPV and influenza A virus while ruling out bacterial and other viral co-infections. This case underscores the diagnostic challenges in differentiating viral from bacterial respiratory infections and highlights the critical role of incorporating molecular testing into routine clinical workflows for community-acquired pneumonia. Multiplex PCR panels are essential for the early detection of respiratory pathogens and can guide antimicrobial stewardship efforts by optimizing treatment strategies. However, the use of multiplex PCR panels should be carefully considered and reserved for selected cases, as these assays frequently detect multiple viral pathogens, including in extreme cases or asymptomatic individuals. The clinical significance of such findings must be interpreted within the broader context of the patient’s condition, as the mere presence of viral nucleic acids does not necessarily indicate active infection or causality.

PCR is highly sensitive to detecting respiratory viruses but has notable limitations, including false positives and the detection of viruses in asymptomatic individuals. Serology provides an additional diagnostic yield by identifying immune responses to infections, thereby improving the accuracy of viral pneumonia diagnoses [21].

In our case, serologic testing for respiratory viruses was not performed, as it is not routinely conducted in our laboratory. The detection of HMPV by RT-PCR in the upper respiratory tract has been frequently reported using the results for a clinical diagnosis in the absence of serologic confirmation [22,23]. The EPIC study, which aimed to determine the etiology and burden of community-acquired pneumonia in the United States, highlighted that serology increased the diagnostic yield for all viral infections as a reliable tool [21].

Chest CT findings in our patient revealed bilateral ground-glass opacities, consistent with prior retrospective studies involving both immunocompetent and immunocompromised adult inpatients with HMPV infection. These studies found that the most frequent clinical diagnoses associated with HMPV infection were pneumonia, acute bronchitis, and acute exacerbation of chronic obstructive pulmonary disease, with ground-glass opacities being the predominant CT abnormality [24]. Currently, treatment for HMPV infection remains primarily supportive, as no specific antivirals have been licensed. However, two potential therapeutic options under investigation include ribavirin and intravenous immunoglobulin. Similarly, no vaccines for HMPV are currently available, although substantial efforts have been made to develop an effective vaccine [1,7,25].

## 4. Limitations

While the detection of influenza A virus and HMPV in our patient suggests their potential pathogenic role, their direct involvement in lung pathology, respiratory dysfunction, and CT findings of our patient cannot be definitively established based on molecular testing alone. It is well recognized that multiplex PCR panels frequently identify viral pathogens in the upper and middle airways, even in asymptomatic individuals, and their mere presence does not necessarily confirm causality. To determine whether pneumonia is caused by influenza A virus and HMPV co-infection, or by one of these viruses alone, histopathological evidence of viral foci within lung tissues will be required. However, in clinical practice, the combination of a compatible clinical presentation, the absence of bacterial pathogens, and a lack of response to antibiotics often supports a viral etiology, even in the absence of histopathological confirmation.

## 5. Conclusions

HMPV and influenza virus infections can lead to severe pneumonia with respiratory failure in individuals with HIV, especially in cases of co-infection. Molecular testing of respiratory samples is crucial for rapid diagnosis, facilitating appropriate management while preventing unnecessary antibiotic use or escalation to broad-spectrum antibiotics in cases of worsening respiratory symptoms. However, multiplex PCR panels may identify viral pathogens in the upper and middle airways, even in asymptomatic individuals, and their only presence does not necessarily confirm causality. Therefore, their use should be carefully and critically interpreted considering the clinical picture and all other microbiological tests performed.

## Figures and Tables

**Table 1:** Patient’s examinations upon admission to infectious disease unit.

Laboratory Analysis	Patient’s Result	Reference Range
WBC (cells/μL)	8800	4000–11,000
Neutrophils (cells/μL)	7300	4000–7400
Lymphocytes (cells/μL)	950	2000–4800
Monocytes (cells/μL)	690	160–1000
PLT (n/μL)	163,000	150,000–450,000
Hb (g/dL)	15.1	12–16
ALP (U/L)	98	35–104
ALT (U/L)	20	0–35
AST (U/L)	13	0–35
GGT (U/L)	19	5–36
Sodium (mmol/L)	136	132–146
Potassium (mmol/L)	4.8	3.7–5.4
Creatinine (mg/dL)	1.86	0.51–0.95
CRP (mg/L)	191	<5

WBC: white blood count, PLT: platelets, ALP: alkaline phosphatase, ALT: alanine transaminase, AST: aspartate aminotransferase, GGT: gamma-glutamyl transferase, CRP: C-reactive protein.

## Data Availability

Data will be made available upon request.

## Abbreviations

The following abbreviations are used in this manuscript:

ABG	Arterial blood gas
CRP	C-reactive protein
CT	Computed tomography
HMPV	Human metapneumovirus
HFNC	high-flow nasal cannula
PLWH	people living with HIV
